# Supplementary material for: A Global Screen for Assembly State Changes of the Mitotic Proteome by SEC-SWATH-MS
Source: Cell Syst. 2020 Feb 26;10(2):133–155.e6. doi: 10.1016/j.cels.2020.01.001 (PMC7042714; doi:10.1016/j.cels.2020.01.001)

Q9Y6I9 | TX264_HUMAN | TEX264 ZSIG11 UNQ337/PRO536
Monomer MW [kDa]: 34.189 Monomer expected elution fraction: 50

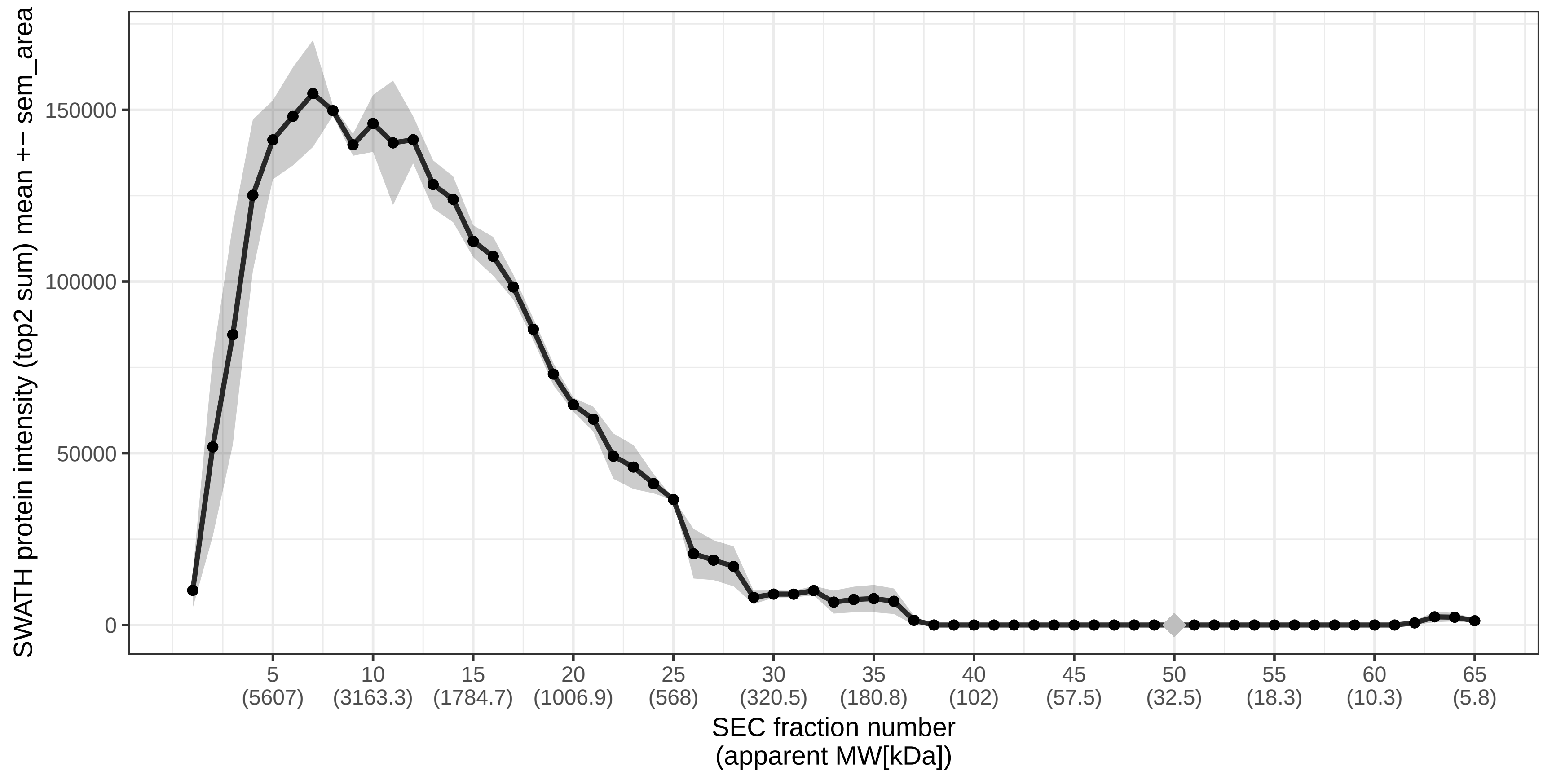

Supplement: Data S1. SEC-SWATH-MS Protein Chromatograms, Related to Figure 1 [file mmc6.zip › SECchrom_Q9Y6I9_TX264_HUMAN_TEX264_ZSIG11_UNQ337_PRO536.pdf]
